# HOME-BASED CARE AND PATIENT OUTCOMES: DOES THE DELIVERY TEAM MATTER?

**DOI:** 10.1093/geroni/igad104.0120

**Published:** 2023-12-21

**Authors:** Norma Coe, Chuxuan Sun, Courtney Van Houtven, Anirban Basu, R Tamara Konetzka

**Affiliations:** University of Pennslyvania, Philadelphia, Pennsylvania, United States; University of Pennslyvania, Philadelphia, Pennsylvania, United States; Duke University School of Medicine, Durham, North Carolina, United States; University of Washington, Seattle, Washington, United States; University of Chicago, Chicago, Illinois, United States

## Abstract

A major challenge facing the U.S. is how to best to meet the growing demand for person-centered, high-quality long-term care in the least restrictive setting possible for community-dwelling older adults who need assistance with daily activities. This paper asks whether who is providing home care impacts patient outcomes. The relative benefits to the care recipient of informal care, formal care, or a combination of the two is largely unknown. A systematic review (Coe et al. 2021) found informal care, either solo or in concert with formal care, tends to lead to improvements in health and well-being of the care recipient compared to formal care alone. However, little of that research can show causation, and most is not based in the U.S. Recipients’ utilization of formal and informal care is affected by factors that also may affect outcomes, complicating the estimation of any causal relationship. Using the 2002-2018 Health and Retirement Study (HRS), we use family structure variables as instruments for both formal care and the combination of formal and informal care to estimate the causal impact of the home health care team on self-reported mental and physical health outcomes. Once the endogeneity of the care team is accounted for, having both formal and informal care leads to higher self-reported health and having only formal care leads to worse mental health outcomes, such as higher depression and lower positive affect, compared to informal care alone. Mobility measures seem unaffected by the type of home care provider.

